# Clinical, parasitological and molecular profiles of Cutaneous Leishmaniasis and its associated factors among clinically suspected patients attending Borumeda Hospital, North-East Ethiopia

**DOI:** 10.1371/journal.pntd.0008507

**Published:** 2020-08-25

**Authors:** Habtye Bisetegn, Ayalew Jejaw Zeleke, Endalamaw Gadisa, Girma Shumie, Demekech Damte, Tiruework Fenta, Sinkinesh Behaksra, Abebe Genetu Bayih

**Affiliations:** 1 Department of Medical Laboratory science, College of Medicine and Health Sciences, Wollo University,Dessie, Ethiopia; 2 Department of Medical Parasitology, School of Biomedical and Laboratory Sciences, University of Gondar, Gondar, Ethiopia; 3 Armauer Hansen Research Institute (AHRI), Addis Ababa, Ethiopia; National Institutes of Health, UNITED STATES

## Abstract

**Background:**

Cutaneous leishmaniasis is one of the most neglected tropical diseases increasing in its public health importance. In Ethiopia over 28 million people are living at risk of infection.

**Method:**

Institution based cross-sectional study was conducted at Borumeda Hospital from February to May 2019. A total 205 leishmaniasis suspected patients were included by systematic random sampling technique. Socio demographic characteristics were collected using pre-tested questionnaires. Parasitological investigation was done from skin slit sample by using Geimsa staining method. Species identification was done by PCR-RFLP. Data were entered in to EpiData version 3.1 and analyzed using SPSS version 20 software. P-value of ≤ 0.05 was considered as statistically significant.

**Result:**

A total of 205 participants consisting 59% male and 41% female included in this study. The mean age (±SD) of the study participants was 31.9 (±14.29). The overall prevalence of cutaneous leishmaniasis was 22.4% (46/205). The prevalence in males (13.7%) was higher than in females (8.8%). It was more prevalent in the age group 16-45years old (15.6%). Clinically, 60% of patients’ hade single lesion with 1.55 average number of lesions. About 30.7% of patients’ had indurated plaque type of lesion. Most of the lesions were found on head and face (59%). House near to farmland, presence of hyrax in the village and presence of other cutaneous leishmaniasis cases in the neighborhood were independent predicator of cutaneous leishmaniasis prevalence. *L*.*aethopica* was found to be the etiologic agent of cutaneous leishmaniasis in the study participants.

**Conclusion:**

The prevalence of cutaneous leishmaniasis was 22.4%, this alerts the need of intervention. It is statistically associated with house near to farm land, presence of other cutaneous leishmaniasis cases in the neighborhood and presence of hyrax in village. Head and face were the most common sites of lesion.

## Author summary

Cutaneous leishmaniasis (CL) is vector-born intracellular protozoan infection of human. It is the most common form of leishmaniasis. Cutaneous leishmaniasis imposes a major public health problem to human being. Cutaneous leishmaniasis is associated with a wide spectrum of clinical presentation like nodular, Papular, nodulo-Papular, diffuse induration, nodulo-ulcerative and indurated plaque. Its clinical manifestation usually begins with an erythematous papule seen at the site of inoculation, then papule enlarges and breaks, forming a painless ulcer with a well-demarcated raised border with different centimeters in diameter, finally this will end up with depressed scar after healing. The lesions usually occur in the face and exposed extremities. Even though CL is not fatal, it can lead to disfigurement and lifelong scar leading to social exclusion and stigmatization. The number of cases and epidemiological foci of CL in Ethiopia is increasing. Here we confirm endemicity of CL in Kutaber district. We also showed as CL is associated with the presence of hyrax, working in and near to farm land and the existence of CL lesion in the neighbor.

## Introduction

Leishmaniasis is vector-borne neglected tropical disease caused by obligate intracellular protozoan parasite of the genus *Leishmania* [1]. The infection is transmitted to human by the bit of female sand fly of the genus *Phlebotomus* in the Old World and *Lutuzomiya* in the New World [2]. It is increasing in its geographical distribution being endemic in Asia, Africa, Mediterranean regions and America. About 1.5 to 2 million new cases occurred annually in the globe with 350 million people being at risk of infection. It leads to a death of 70,000 people annually [3]. Clinically, leishmaniasis can be cutaneous leishmaniasis (CL), mucocutaneous leishmaniasis or visceral leishmaniasis [4]. Cutaneous leishmaniasis is the most common form of leishmaniasis with 0.7 to 1.2 million annual new cases globally [5].

Factors involved in the emergence and Worldwide spread of leishmaniasis include change in temperature, habits of irrigation, deforestation, climatic change, and immune suppression by different immune suppressants, existence and increment in drug resistance, traveling to endemic regions and dog importation, war, poor socioeconomic status and low household level [6].

In Ethiopia CL was first described in 1913 by Italian scientist Martoglio F, who indicated as the CL was locally adopted and common in the highlands of Ethiopia. A study conducted in 1973 reported the existence of the diseases and its distribution in different parts of Ethiopia [7]. In Ethiopia, currently around 29 million people are at risk of infection [8]. Ethiopia had around 20,000 to 50,000 estimated new cases of CL every year [9]. The parasites that cause CL in Ethiopia are mainly *L*. *aethiopica* and rarely *L*. *major* [10]. Cutaneous leishmaniasis is endemic in different regions of Ethiopia such as in Tigray national regional state around Mekele city, Saesie Tsaeda-emba district Eastern Tigray and Southern Nations and Nationalities people’s regional state (SNNPR) around Silte Zone [11–13]. Estimating the burden of CL is still challenging due to its clinical and epidemiological diversity, geographical clustering and absence of reliable surveillance data [14]. The absence of information indicating population based prevalence, incidence, risk factors and its impact on socioeconomic profile are still the main gaps in determining CL burden [15]. Although cases are frequently reported in the health institutions, there was paucity documented data about CL in the study area Northeast Ethiopia. Therefore, this study was conducted to provide documented evidence on clinical, parasitological and molecular profiles of cutaneous leishmaniasis and its associated factors among patients attending Borumeda Hospital North-East Ethiopia.

## Materials and methods

### Ethical approval and consent to participate

Ethical clearance was obtained from ethical review committee of School of Biomedical and Laboratory Sciences, College of Medicine and Health Sciences, University of Gondar. Written consent was obtained from each study participants. A written signed assent was also obtained from each child Participant’s parent or guardians on the child’s behalf. Positive patients received the appropriate treatment according to the Hospital treatment guideline.

### Study design and area

Institution based cross-sectional study was conducted at Borumeda Hospital North-east Ethiopia from February to May, 2019. The Hospital is found at 411 km North-east of Addis Ababa, capital of Ethiopia. It gives service primarily in Dermatology and Ophthalmology outpatient department to patients from Dessie town and surrounding areas. Majority of the patients came from Kutaber District which is found at 11 km from the Hospital. The area of the district is about 719.92 km^2^ and had 103,489 population sizes. Kutaber area is typical plateau at 2650m above sea level and in defile position to the north and south of which steep slopes rise to 3,000m, then climb more gradually to summits around 3,400m. To the East is a wide alluvial valley frequently flooded, but providing lush pasture as it is dried. To the West the land drops suddenly in to tributary gorge to the Blue Nile, down to 2,200m [7].

### Eligibility criteria

All CL suspected patients who visited Borumeda Hospital dermatology outpatient department (OPD) were included. Cutaneous leishmaniasis patients who were on treatment and follow up were excluded.

### Sample size and sampling method

The sample size of this study was determined by single population proportion formula using 14% prevalence reported by study conducted in SasieTsaeda-emba district, Eastern Tigray, Northern Ethiopia [12]. A 95% confidence interval, 5% margin of error and 10% of non-response rate were used. A total of 205 participants were recruited using systematic random sampling technique.

### Data collection

Socio-demographic characteristics and risk factors were collected using pre-tested questionnaires. Clinical characteristics of the lesions were assessed by experienced dermatologists. After disinfecting the lesion with 70% alcohol from inside to outside, skin slit was taken from the edge of lesions using single use surgical blade. Thin smear were prepared on a clean microscopic slide. A smear was allowed to air dry and then fixed with absolute methanol. Finally it was stained with 10% Geimsa stain. Slides were examined microscopically to detect the amoastigote stage of *Leishmania* parasites.

### *Leishmania* culture

After cleaning the lesion with 70% alcohol, skin slit was taken and placed into the Nicoll-Novy -MacNeal (NNN) media that contains 2mL Lock’s solution and incubated in a 26–28°C incubator. Growth was detected by observing the promastigote microscopically. Positive media were transported to Armauer Hansen Research Institute (AHRI) Addis Ababa Ethiopia for species typing.

### Molecular identification of the parasite by PCR

#### DNA preparation

Target DNA was extracted from cultured promastigote. The over lay of each culture positive tube was transferred in to separate falcon tube and centrifuged for 10 minutes. The sediment was washed 3 times with phosphate buffer saline (PBS) and the pellet was treated with lysis buffer (10Mm Tris-HCl (pH 8), 5M EDTA (PH 8), 5M NaCl and 10% SDS (Sodium dodecyl sulfate). The lysate was transferred to a clean eppendroff tube, then, 20 microliter Proteinase K was added and incubated overnight and phenol chloroform isoamyl alcohol (25:24:1 PCIA) extraction was carried out as described elsewhere [16,17].

#### ITS1-PCR amplification of Leishmania isolate

The ITS1 of leishmania isolate was amplified using pair of primers (forward prime LITSR (5’ CTGGATCATTTTCCGATG 3´ and revers primer L5.8S (5’ TGATACCACTTATCGCACTT 3’)) with PCR condition described elsewhere [18–20]. The product of the PCR amplification was tested using electrophoresis with 1% agarose gels in 1x TBE, finally visualized by UV light after staining with ethidium bromide.

#### Restriction analysis

The ITS1-PCR products were digested with restriction enzyme HaeIII prototype according to the manufacturer’s instruction. The restriction fragments were analyzed by gel electrophoresis/ ethidium bromide staining at 120V in 1×TAE buffer in agarose gel. The result was visualized by UV light.

### Data quality control

To assure the quality of the data, the questionnaires were prepared in English and translated to Amharic and back translated. The questionnaires were adopted from published research and pretested. Training was given to data collectors. Microscopic examination was carried out by two laboratory professionals and the result was confirmed by third laboratory professional and there was no discrepancy. All material used for media preparation were sterilized by autoclaving at 121°C for 20 minutes. In culture media preparation, media from each batch was incubated and checked for sterility. Negative extraction was used to control contamination during DNA extraction process. Distilled water and previously confirmed cases were used as negative and positive control respectively.

### Statistical analysis

Data were entered in to EpiData version 3.1 and exported and analyzed using SPPS version 20 software package. Univariate and multivariate logistic regression were used to evaluate the existence and strength of association between dependent and predictor variables. The 5% level of significance and 95% confidence interval were used. P-value of ≤ 0.05 was considered as statistically significant.

## Result

### Socio-demographic characteristics of the study participants

A total 205 participants were included in this study. Most of the study participants were male (59%). The age distribution of the study participant was 2–73 years old. The mean age and standard-deviation of the study participants were 31.9 and ±14.29. Out of 205 study participants about 60% of them were rural residents (Table 1).

**Table 1:** Demographic characteristics of patients at Borumeda Hospital, Northeast Ethiopia 2019.

Variables		Frequency	Percentage
**Sex**	Male	121	59
	Female	84	41
**Age in years**	1–15	20	9.8
	16–45	144	70.2
	≥46	41	20
**Educational Status**	Illiterate	80	39
	Primary	68	33.2
	Secondary	29	14.1
	College and above	28	13.7
**Residence**	Urban	82	40
	Rural	123	60
**Occupational status**	Farmer	55	28.6
	House wife	51	24.9
	Government institution worker	20	9.8
	Private worker	19	9.3
	Searching for work	16	7.8
	Student	44	21.5

### Clinical characteristics of the lesions

About 59% of the study participants had lesions on their head and face and 18% of them had lesion on the upper limb. About 60% of the patients had single lesion while 30.2% of them had two lesions. The average number of lesion was 1.55. The most frequently observed type of lesion was indurated plaque (30.7%) followed by nodular (17.1%), popular (14.1%), diffuse induration (13.7%), nodulo-popular (12.2%) and nodulo-ulcerative (12.2%). Around 52.7% of patients had lesion about 6–12 month old and 39% had lesions less than six months.

### Prevalence cutaneous leishmaniasis

The overall prevalence of CL was 22.4% (46/205). The prevalence in males was 13.7%. The age group between 16–45 years old showed 15.6% prevalence. The prevalence of CL was 15.6% in the rural and 6.8% in the urban resident (Table 2).

**Table 2:** Distribution of CL by demographic characteristics at Borumeda Hospital, Northeast Ethiopia 2019.

Demographic characteristics		Smear positive Number/ (%)	Smear negative Number/ (%)
** Age group**	1–15	4(2)	16(7.8)
	16–45	32(15.6)	112(54.6)
	46 and above	10(4.9)	31(15.1)
** Sex**	Male	28(13.7)	93(45.4)
	Female	18(8.8)	66(32.2)
**Educational status**	Illiterate	17(8.3)	63(30.7)
	Primary	14(6.8)	54(26.3)
	Secondary	9(4.1)	20(9.8)
	College and above	6(2.9)	22(10.7)
**Residence**	Rural	32(15.6)	91(44.4)
	Urban	14(6.8)	68(33.2)
**Occupation**	Farmer	12(5.9)	43(21)
	Housewife	10(4.9))	41(20)
	Gov.t institutions	6(2.9)	14(6.8)
	Private institutions	1(0.5)	18(8.8)
	Searching for work	6(2.9)	10(4.9)
	Student	11(5.4)	23(16.1)
**Outdoor activities**	Yes	32(15.6)	75(36.6)
	No	14(6.8)	84(41)
**House near to farm land(300m)**	Yes	29(14.1)	49(23.9)
	No	17(8.3)	110(53.7)
**Presence of hyrax**	Yes	31(15.1)	32(15.6)
	No	15(7.3)	127(62)
**Presence of gorge**	Yes	36(17.6)	69(33.7)
	No	10(4.9)	90(43.9)
**Presence of CL lesion in neighbor**	Yes	36(17.6)	74(36.1)
	No	10(4.9)	85(41.5)

### Prevalence of CL by clinical characteristics

About 13.7% of *Leishmania* prevalence was from participants who have lesion on their head and face. From the total 46 *Leishmania* positive patients, about 23.9% of them had indurated plaque types of lesion. The prevalence of CL was highest in patients with lesions less than 12 months old (12.2%), while lesion greater than 12 months had the least prevalence (1.5%) (Table 3).

**Table 3:** Cutaneous leishmaniasis prevalence and clinical profiles of the study participant patients attending at Borumeda Hospital, South Wollo, Dessie, Ethiopia 2019.

Variable		Smear positive Number/ (%)	Smear negative Number/ (%)
**Site of lesion**	Head and face	28(13.7)	93(45.4)
	Upper limb	8(3.9)	29(14.1)
	Lower limb	6(2.9)	11(5.4)
	Neck	2(1)	11(5.4)
	More than one site	2(1)	15(7.3)
**Type of lesion**	Nodulo-ulcerative	10(4.9)	15(7.3)
	Nodulo- Papular	9(4.4)	16(7.8)
	Indurated plaque	11(5.4)	52(25.4)
	Diffuse induration	3(1.5)	25(12.2)
	Nodular	8(3.9)	27(13.2)
	Papular	5(2.4)	24(11.7)
**Number of lesion**	One lesion	27(13.2)	96(46.8)
	Two lesion	16(7.8)	46(22.4)
	Three lesion and above	3(1.5)	17(8.3)
**Duration of lesion**	<6 months	25(12.2)	55(26.8)
	6–12 months	18(8.8)	90(43.9)
	>12 months	3(1.5)	14(6.8)

### Univariate and multivariate logistic regression of CL prevalence with demographic exposure related risk factors

In Univariate analysis independent variable such as working in and near to farmland, house near to farmland, presence of gorge in the village, presence of hyrax in the village and the presence of CL lesion were found to be statistically associated with CL prevalence (p-value <0.05). In multivariate analysis, house near to farmland, presence of hyrax in the village and presence of CL lesion were remained as an independent predicators of cutaneous leishmaniasis prevalence (p-value< 0.05) (Table 4).

**Table 4:** Univariate and multivariate analysis of predicator variables to the prevalence of cutaneous leishmaniasis among clinically suspected patients attending at Borumeda Hospital, Northeast Ethiopia 2019.

Demographic and exposure related risk factors		COR(95%CL)	p-value	AOR(95%CL)	P-Value
**Residence**	Rural	1.71(0.85–3.45)	0.135	.308(0.17–1.77)	.308
	Urban	1		1	
**Outdoor activities**	Yes	2.56(1.27–5.16)	.009	2.02(0.73–5.6	0.177
	No	1			
**House near to farm land**	Yes	3.83(1.93–7.61)	.00	4.66(1.57–13.89)	.006
	No	1			
**Presence of hyrax**	Yes	8.2(3.96–16.99)	00	6.14(2.13–17.7)	.001
	No	1			
**Presence of gorge**	Yes	4.69(2.18–10.12	00	2.68(0.9–7.99)	.076
	No	1			
**CL lesion in neighbor**	Yes	4.1(1.92–8.9)	00	7.03(2.47–20.05)	.000
	No	1			
**Type of lesion**	Nodulo-ulcerative	3.2(0.92–11.12)	.069	4.472(0.85–23.56)	.077
	Nodulo- papular	2.7(0.76–9.55)	0.123	2.52(0.48–13.38)	0.277
	Indurated plaque	1.02(0.32–3.250	0.979	.83(0.17–4.19)	0.823
	Diffuse induration	0.57(0.12–2.67)	0.482	0.56(0.08–3.77)	0.55
	Nodular	1.42(0.41–4.94)	0.579	1.86(0.33–10.69)	0.485
	Papular	1		1	
**Duration of lesion**	<6 months	2.12(0.56–8)	0.269	7.3(0.96–55.96)	.055
	6–12 months	0.92(0.24–3.5)	.920	1.96(0.28–13.99)	0.5
	>12 months	1		1	

### Molecular characterization of *Leishmania* parasites in the study area

#### ITS1-PCR *Leishmania* isolates

**Fig 1:**
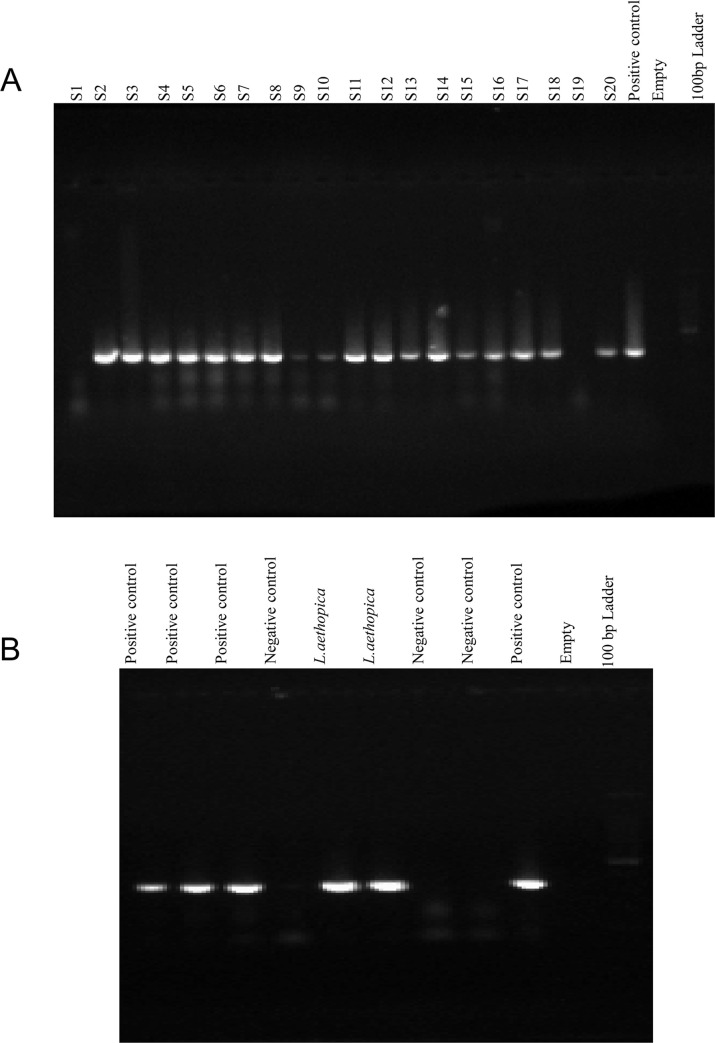
Gel electrophoresis of PCR products after staining with ethidium bromide. Lane S1 up to S20 is sample (the sample in lane S1 was almost null), L. *aethiopica* as a positive control, distilled water as negative control and 100bp DNA ladder.

DNA was extracted from cultured promastigote of 20 samples using phenol-chloroform extraction method. Amplification of ITSR1 ribosomal DNA was performed using forward primer (LISTR) and revers primer (L5.8S). Gel electrophoresis of the PCR product showed around 350 base pair of *Leishmania* isolates both in the sample taken from the cultured promastigote and *L*.*aethopica* reference strain (Fig 1).

#### Restriction fragment length polymorphism (RFLP)

**Fig 2:**
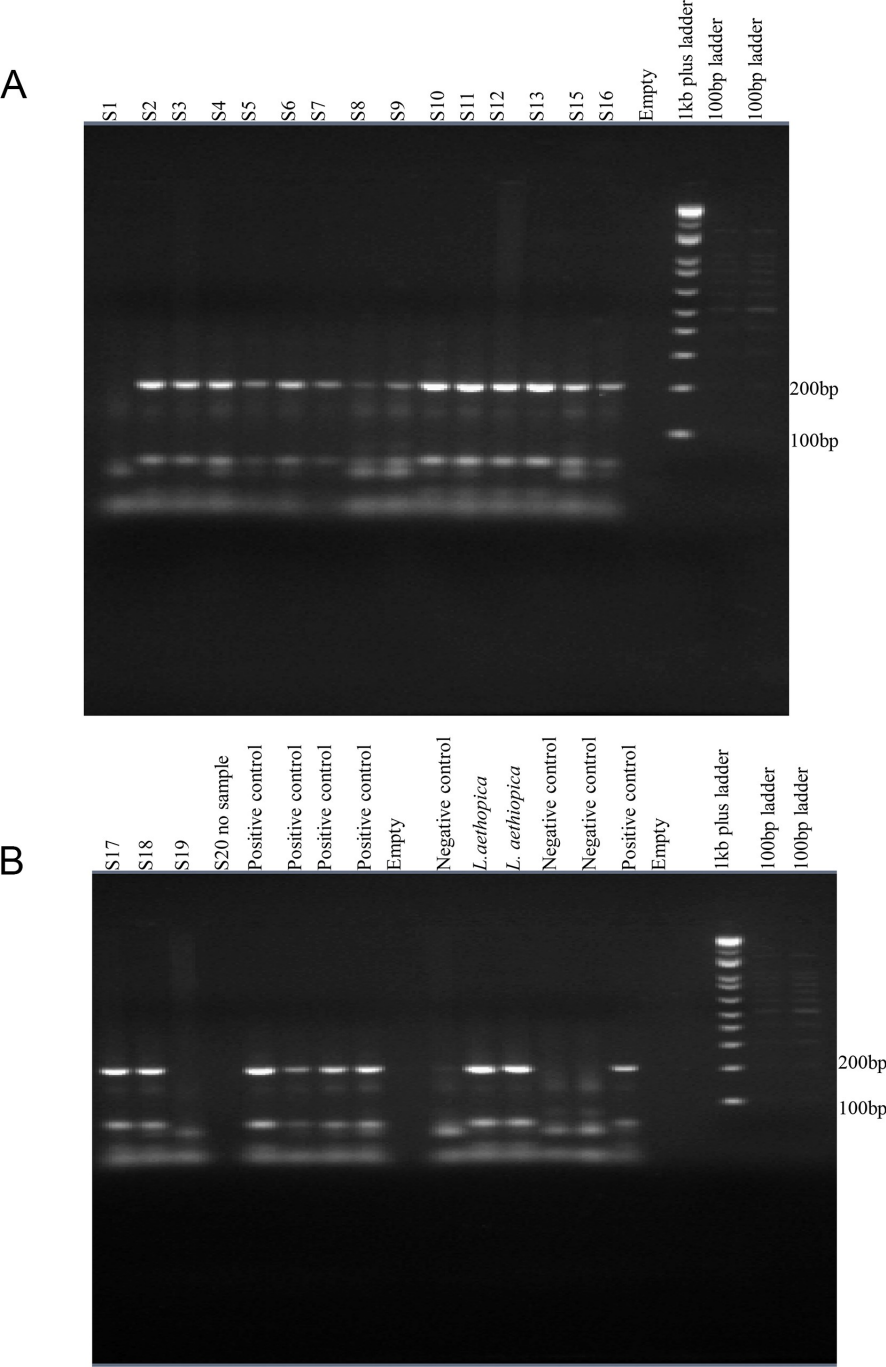
Representative picture for HaeIII digestion of internal transcribed spacer (ITS1) for species typing of leishmania isolate from Borumeda Hospital Ethiopia. In the restriction fragment length polymorphism (RFLP) analysis lane S1-S20 are samples (in lane S1, S19 and S20 the sample was insufficient). Distilled water as negative control, *L*.*aethopica* reference strain and 1kb plus and 100bp DNA ladder.

PCR products were digested using restriction enzyme endonuclease HaeIII at 37 ^o^_C_ for three hours. Gel electrophoresis of the digested product showed that all the samples produced visible bands of approximately 200bp and 56bp which was similar to bands of *L*. *aethiopica* reference strains. This finding confirmed that the causative agent of CL in the study area is *L*.*aethopica* (Fig 2).

## Discussion

Cutaneous leishmaniasis is endemic in different parts of Ethiopia and reported since 1913 [7]. It is still a major cause of morbidity and disfigurement. About 28 million people in Ethiopia live in area of active transmission [8]. In this study the prevalence of CL was 22.4%, this finding was higher than reports from Ethiopia, in Sasie Tseda Emba district Ethiopia (14%), Silti district (4.8%), Mekele city (5.6%) [11–13]. It was also higher than reports from Yemen (18.87%) and Iran (4.7%) [21,22]. This difference might be due to the presence reservoir hosts in the study area, environmental factors, climatic factors, landscape, entomological factors, and outdoor activities. On the other hand, this finding was lower than studies conducted in Ochollo, South-west Ethiopia (64.8%), Libya (43%), Yemen (74.1%), North-west Yemen (96.23%), Sri Lanka (41.5%), Erbil Iraq (70.6%), Pakistan (50.8%) and Colombia (79.1%) [23–30]. The low prevalence in this study might be due to use of traditional drugs, applying of hot objects on the lesion and variation in environmental and behavioral factors.

The prevalence was higher in male than female (13.7% vs 8.8%) this finding agreed with the finding from Sasie Tsaeda Emba district, Ethiopia, Northern Ethiopia, Sri-Lanka and Pakistan [11,12,27,29]. The high prevalence of CL in male participants might be due to; outdoor activity of male and male usually wear shorts which fit for agricultural activities and do not wear shirts while sleeping during the warm season this helps the sand fly to bit them easily. In contrast, females are usually restricted at home and they wear cloth that covers most of their body.

Even though all age groups are affected by CL, the highest prevalence was in the age group 16–45 years old. This finding agreed with the finding of studies conducted in Mekele Northern Ethiopia, Sri Lanka and Mazandaran Province, Iran [11,31,32]. The high prevalence of CL in the age group 16–45 might be because of this age groups are active working force, which had out door exposure and had high probability of traveling to leishmaniasis endemic areas.

Rural residents were more affected than urban residents, this was supported by the finding from western Islamic Republic of Iran [33]. This could be due to rural people are engaged in agricultural activities and *leishmania* parasite reservoir hosts are very common in rural areas than cities and towns. The prevalence was also higher in farmers (5.9%) as compared to other occupation; this might be due to high chance of farmers to be exposed to sand fly during farm work. Cutaneous leishmaniasis prevalence was highest in samples taken from lesion less than 6 month old. This agrees with the report from Sri Lank which had reported 54.5% of the smear positive lesion were lasted in less than 6 months [27].

The average number of lesion was 1.55 per person with a maximum of 6 lesions. In contrast, a study in Pakistan reported an average of 1–11 lesion with amaximum of 11 lesions [29] and in western Islamic Republic of Iran reported as the average number of lesion was 1.8 with maximum number of lesion being 7 [33]. The highest duration of lesion in this study was about 6–12 month old (52.7%) this difference may be associated with secondary bacterial infection, immune response of the patient, life style of the patient and strain of the parasite.

In the present study house near to farmland, presence of hyrax in the village and presence of CL lesion showed statistically significant association with the prevalence of cutaneous leishmaniasis after adjusting other risk factors. This finding was similar to the finding reported Mekele city, Ayder referral hospital, and studies in Sasie Tseda Emba district Northern Ethiopia [11,12].

Working in and near to farmland, house near to farmland, presence of gorge in the village, presence of hyrax in the village and the presence of CL lesion were statistically associated with prevalence of CL in Univariate logistic regression. However, after adjusting for confounders only house near to farmland, presence of hyrax and CL lesion remain independent predictors of CL prevalence in the study area. The odds of being infected with CL in patients having CL lesion in neighbor and presence of hyrax in the village was seven and six times more likely than their counter reference group respectively (AOR: 7.03(2.47–20.05), 6.14(2.13–17.7)) respectively. Participants whose house is near to farm land are about 4.6 times more likely to be infected with CL (AOR: 4.66(1.57–13.89)).

Cutaneous leishmaniasis is endemic the study area. Male were infected in higher proportion than females because of out-door activity of male, wearing style of and other social and occupational factors. Age group 16–45 is the most affected age groups due to high possibility of contact with sand fly, high tendency of travel to different endemic areas. House near to farm land, presence of CL lesion in neighbor and presence of hyrax were independent predicator of CL prevalence. During community mobilization majority of the community are not aware of the existence of treatment for CL in hospital. *L*.*aethopica* is confirmed to be etiologic agent of CL in the study area.

The authors recommend the ministry of health, regional, zonal and district health office to recognize the endemicity of the disease in the study area and work together to establish well organized treatment and advanced diagnostic center as well as work on the community to create awareness about the causative agent, mode of transmission and source of infection.
